# Quantifying the therapeutic requirements and potential for combination therapy to prevent bacterial coinfection during influenza

**DOI:** 10.1007/s10928-016-9494-9

**Published:** 2016-09-27

**Authors:** Amber M. Smith

**Affiliations:** Department of Infectious Diseases, St. Jude Children’s Research Hospital, Memphis, TN 38105 USA

**Keywords:** Kinetic modeling, Influenza, Pneumococcus, Coinfection, Antivirals, Antibiotics, Immunotherapy, Combination therapy

## Abstract

Secondary bacterial infections (SBIs) exacerbate influenza-associated disease and mortality. Antimicrobial agents can reduce the severity of SBIs, but many have limited efficacy or cause adverse effects. Thus, new treatment strategies are needed. Kinetic models describing the infection process can help determine optimal therapeutic targets, the time scale on which a drug will be most effective, and how infection dynamics will change under therapy. To understand how different therapies perturb the dynamics of influenza infection and bacterial coinfection and to quantify the benefit of increasing a drug’s efficacy or targeting a different infection process, I analyzed data from mice treated with an antiviral, an antibiotic, or an immune modulatory agent with kinetic models. The results suggest that antivirals targeting the viral life cycle are most efficacious in the first 2 days of infection, potentially because of an improved immune response, and that increasing the clearance of infected cells is important for treatment later in the infection. For a coinfection, immunotherapy could control low bacterial loads with as little as 20 % efficacy, but more effective drugs would be necessary for high bacterial loads. Antibiotics targeting bacterial replication and administered 10 h after infection would require 100 % efficacy, which could be reduced to 40 % with prophylaxis. Combining immunotherapy with antibiotics could substantially increase treatment success. Taken together, the results suggest when and why some therapies fail, determine the efficacy needed for successful treatment, identify potential immune effects, and show how the regulation of underlying mechanisms can be used to design new therapeutic strategies.

**Electronic supplementary material:**

The online version of this article (doi:10.1007/s10928-016-9494-9) contains supplementary material, which is available to authorized users.

## Introduction

Influenza virus infections result in 15–65 million infections, over 200,000 hospitalizations, and over 30,000 deaths each year in the U.S. [32, 45]. This is in part due to the number of antigenically distinct influenza virus strains, the lack of comprehensive strain coverage in the vaccines, and the complications arising from underlying health conditions and/or secondary bacterial infections (SBIs). SBIs, in particular, have accounted for 40–95 % of influenza-related mortality in past pandemics [4, 17, 29, 48]. Vaccines against bacterial pathogens can reduce the coinfection component [15, 25, 27], but their efficacy is limited to the vaccine strains and some bacterial vaccines have reduced effectiveness in influenza virus-infected hosts [25, 27]. Treatment with antimicrobial agents may also improve disease outcome and reduce SBI incidence [7, 8, 12, 13, 18, 21, 26], but many provide only partial protection, have time dependent efficacy, and/or cause adverse effects. Thus, new preventative and therapeutic strategies are needed. These may require utilizing current antimicrobial agents on different time scales and/or exploiting the mechanisms that regulate disease to increase the efficacy of treatment or to develop new targets.

Antivirals for use against the influenza virus slow disease progression and reduce symptoms by preventing new host cells from being infected [9]. However, this does not typically result in a significant reduction in viral burden [1, 18, 21], and the efficacy is reduced if therapy is initiated more than 2–3 days after symptom onset [1, 21]. The decreased efficacy late in infection occurs because the antivirals target stages in the viral life cycle (i.e., infection of cells, and virus replication and production) that are dominant only during the first 2 days post-influenza infection (pii) [40]. Although the duration and severity of the viral infection are not reduced with late antiviral treatment, treatment as late as 5 days pii can slow the progression of pneumonia and decrease SBI mortality [21]. The mechanism(s) underlying this effect are unclear, but the nominal reduction in viral loads may have sufficient downstream consequences on the immune response. Antiviral treatment may also reduce the post-bacteria viral load rebound that is observed during some SBIs [39, 43, 47], which may be due to an increase in virus production/release [43, 46].

Antibiotics directly target the pathogen by causing lysis or by inhibiting protein synthesis, but these drugs have limited effects in coinfected hosts [7, 12, 13, 16]. Lytic antibiotics (e.g., ampicillin) effectively reduce pathogen load during SBIs, but do so at the expense of a robust inflammatory response [7, 12]. In contrast, inhibitory antibiotics (e.g., clindamycin and azithromycin) have reduced bactericidal effects but limit tissue damage and inflammation [12, 13, 16]. Although this class of drugs can provide a clinical cure primarily through their anti-inflammatory effects and are beneficial in treating coinfected animals, the high pathogenic burden is problematic and may lead to drug resistance. Combining a drug that rapidly eliminates bacteria (e.g., ampicillin) with one that has anti-inflammatory effects (e.g., corticosteroid) seems optimal and does reduce immunopathology during severe pneumonia; however, prophylactic use of corticosteroids impairs viral clearance [7].

Because traditional therapeutic agents like antivirals and antibiotics are suboptimal, targeting specific inflammatory pathways may increase the probability of success. However, this approach requires knowledge about the underlying mechanisms of disease. Several factors affect the likelihood of SBI-associated pneumonia developing, including viral and bacterial strains, transmitted dose size, timing of bacterial exposure, and host immune status (reviewed in [3, 20, 24, 33–35, 37]). In addition, different mechanisms are likely involved in the various stages of SBIs, e.g., bacterial invasion, pathogen kinetics, inflammation, and mortality. Therefore, various therapeutic approaches may be possible. To help tease apart the contribution of different mechanisms on bacterial acquisition and pathogen titer trajectories, my colleagues and I developed a kinetic model [38, 43] that suggested bacterial invasion is due to the virus removing the protective effect of alveolar macrophages (AMs) with 85–90 % efficiency at 7 days pii. Although the underlying mechanism was thought to be a functional inhibition mediated by interferon-$\gamma$ [28, 44], another study better identified the kinetics of these cells and found that AMs are depleted during influenza virus infection [8]. Remarkably, these data validated our model predictions and the maximum amount of depletion, which occurs at 7 days pii [8] and corresponds to the greatest lethality [22], matched our parameter estimate of 85–90 % [43]. Because the AM population is tightly connected to early bacterial clearance, therapeutically replenishing the AM population through immunotherapy during influenza virus infection can improve the pathogenic burden and significantly reduce pneumonia [8].

Knowing the model accuracy and the kinetics of AM depletion allowed us to mathematically derive and experimentally validate a nonlinear relationship between bacterial dose/load and AM depletion that regulates bacterial invasion and kinetics during the initial stages of infection [38]. Understanding these dynamics and their regulation with mathematical precision provides important insight into the possibility of using therapeutics to alter each component and the efficacy necessary for the treatment to be successful. That is, therapeutically reducing the bacterial load (e.g., via antibiotics) will have the same result as increasing the number of AMs (e.g., via immune modulatory drugs or by reducing virus with antivirals), but the nonlinearity of the relationship indicates differential and time-dependent therapeutic requirements.

To further understand the viral and bacterial kinetics under therapy, I used mathematical models [41, 43] and published data on the dynamics after therapy in BALB/cJ mice [8, 12, 21] to investigate the efficacy of an antiviral, an antibiotic, and an immune modulatory agent in the prevention and treatment of influenza and influenza-associated SBIs. The models were used to predict how pathogen dynamics would change under each therapy and to quantify the therapeutic benefit for various intervention efficacies and timing, the minimum therapeutic requirement to achieve a clearance or resolution phenotype, and the potential of combination therapy. The results provide insight into the failure of current therapies, the time-scale of the greatest therapeutic benefit, the efficacy of mono-therapy versus combination therapy, the potential immune consequences of some drugs, and the possibility of new therapeutic targets.

## Methods

### Influenza virus infection model

To describe the kinetics of influenza virus infection, a target cell limited model [2] was used. The model tracks populations of susceptible epithelial (“target”) cells (*T*), newly infected cells that are not yet producing virus ($I_1$), infected cells that have undergone an eclipse phase and are producing virus ($I_2$), and free virus (*V*). Target cells become infected with virus at rate $\beta V$. Infected cells ($I_1$) first enter an eclipse phase at rate *k* then transition to produce virus at rate *p*. Productive infected cells ($I_2$) are lost at rate $\delta$ and virus is cleared at rate *c*. Equations (1–4) represent these dynamics and the model parameters are provided in Table 1. The model schematic and fits to viral titers from mice infected with influenza A/Puerto Rico/8/34 (PR8) are shown in Fig. S1.

1 $$\begin{aligned} \frac{dT}{dt}&=-\beta TV \end{aligned}$$

2 $$\begin{aligned} \frac{dI_1}{dt}&=\beta TV - kI_1 \end{aligned}$$

3 $$\begin{aligned} \frac{dI_2}{dt}&=kI_1-\delta I_2 \end{aligned}$$

4 $$\begin{aligned} \frac{dV}{dt}&=pI_2 - cV \end{aligned}$$

### Influenza-pneumococcal coinfection model

To describe the kinetics of influenza-pneumococcal coinfection, a model that couples single infection models for influenza virus (Eqs. (1–4)) [2] and pneumococcus [42] and includes terms that describe their interactions [43] was used. In this model, the pneumococcal population (*P*) is tracked in addition to the four populations in Eqs. (1–4). Bacteria replicate logistically with maximum rate *r* and tissue carrying capacity $K_P$. The model considers the initial interaction between pneumococci and the first arm of the immune system, AMs ($M_A$), which phagocytose bacteria at rate $\gamma_{M_A}f(P,M_A)$ per cell. This rate decreases as the number of pneumococci increases according to $f(P,M_A) = n^xM_A/(P^x+n^xM_A)$, where each AM is able to phagocytose a maximum of *n* bacteria and *x* is the shape parameter that describes the consumption rate of pneumococci. Virus further decreases this clearance rate according to $\hat{\phi }(V)=\phi V/(K_{PV}+V)$. This term drives bacterial invasion [43] and matches the percentage of AM depletion [8, 38]. Once bacteria invade, virus production/release from infected epithelial cells ($pI_2$) is increased by a factor of $\hat{a}(P)=aP^z$. This term drives the viral rebound (Fig. S2) [43], which may result from IFN inhibition as a consequence of bacterial attachment to infected cells [43, 46]. The model also assumes that virus infection increases the tissue carrying capacity by $\psi V$, which may facilitate bacterial adhesion to cells, and that bacteria increase infected cell death by $\mu P$. However, these two effects were shown to have minimal influence on the dynamics [43]. Altering other processes in the model, such as the rates of viral infection ($\beta V$) and clearance (*c*), produced minimal effects on model dynamics. Equations (5–9) represent these dynamics and the model parameters are provided in Table 1. The model schematic and fits of the model to viral and bacterial titers from mice infected 7 days after PR8 with pneumococcal strain D39 are shown in Fig. S2 [43].

5 $$\begin{aligned} \frac{dT}{dt}&= -\beta TV \end{aligned}$$

6 $$\begin{aligned} \frac{dI_1}{dt}&= \beta TV - kI_1 - \mu PI_1 \end{aligned}$$

7 $$\begin{aligned} \frac{dI_2}{dt}&= kI_1-\delta I_2-\mu PI_2 \end{aligned}$$

8 $$\begin{aligned} \frac{dV}{dt}&= pI_2{\left( 1+\hat{a}(P)\right) }-cV \end{aligned}$$

9 $$\begin{aligned} \frac{dP}{dt}&= rP\left( 1-\frac{P}{K_P\left( 1+\psi V\right) }\right) -\gamma_{M_A} f(P,M_A^*) M_A^*P\left( 1- \hat{\phi }(V) \right) \end{aligned}$$

### Model simulations and parameters

MATLAB ordinary differential equation (ODE) solver (*ode45*) was used to simulate all equations. The parameter values used in this study are given in Table 1 or are stated in the text. The influenza model parameters were obtained by fitting Eqs. (1–4) to viral titer data from individual mice infected with 100 TCID$_{50}$ (50 % tissue culture infectious dose) PR8 [41]. The pneumococcal model parameters were obtained by matching Eq. (9) with $V=0$ to bacterial titer data from individual mice infected with $10^4$, $10^5$, or $10^6$ colony forming units (CFU) pneumococcal strain D39 [42]. The coinfection model parameters were obtained by fitting Eqs. (5–9) to viral and bacterial titer data from individual mice infected with 100 TCID$_{50}$ PR8 followed by 1000 CFU D39 at 7 days pii [43]. The coinfection model and parameters also matched the bacterial titer data from mice infected with pneumococcal strain A66.1 [43], which is the strain used in the studies described below.

**Table 1:** Parameter values of the influenza virus infection model (Eqs. (1–4)) [41], the pneumococcal model (Eq. (9) with $V=0$) [42], the coinfection model (Eqs. (5–9)) [38, 43], and under therapy with antimicrobial agents.

Parameter	Description	Value	Units
*Influenza A virus*			
$\beta$	Virus infectivity	2.8$\,\times\,10^{-6}$	$(\mathrm {TCID}_{50})^{-1}\,{{\text{day}}^{-1}}$
*k*	Eclipse phase	4.0	day^−1^
$\delta$	Infected cell death	0.89	day^−1^
*p*	Virus production	25.1	$(\mathrm {TCID}_{50})\,{{\text{day}}^{-1}}$
*c*	Virus clearance	28.4	day^−1^
*T*(0)	Initial uninfected cells	$10^7$	cells
$I_1(0)$	Initial infected cells	0	cells
$I_2(0)$	Initial infected cells	0	cells
*V*(0)	Initial virus	2.0	$\mathrm {TCID}_{50}$
*Pneumococcus*			
*r*	Bacterial growth rate	27.0	day^−1^
$K_{P}$	Carrying capacity	2.3$\,\times\, 10^{8}$	CFU
$\gamma_{M_A}$	Phagocytosis rate	1.35$\,\times\, 10^{-4}$	$\mathrm {cell}^{-1}\,{{\text{day}}^{-1}}$
*n*	Maximum bacteria per AM	5.0	$\mathrm {(CFU)cell}^{-1}$
*x*	Nonlinearity in $f(P,M_A)$	2.0	
$M_A^*$	Number of AMs	$10^6$	cells
$P_0$	Initial bacteria	See text	CFU
*Coinfection*			
$\phi$	Decrease in phagocytosis rate	0.87 (7 days), 0.646 (3 days)	
$K_{PV}$	Half-saturation constant	1.8$\,\times\, 10^3$	$\mathrm {TCID}_{50}$
*a*	Increase in virion production/release	$1.2\,\times\, 10^{-3}$	$\mathrm {(CFU)}^{-z}$
*z*	Nonlinearity of virion production/release	0.50	
$\psi$	Increase in carrying capacity	1.2$\,\times\, 10^{-8}$	$(\mathrm {TCID}_{50})^{-1}$
$\mu$	Toxic death of infected cells	5.2$\,\times\, 10^{-10}$	$\mathrm {(CFU)}^{-1}$
*Therapy*			
$\varepsilon_{v}$	Efficacy of antiviral treatment	See text	
$\varepsilon_t$	Rate of target cell protection by antivirals	0.68	day^−1^
$\varepsilon_g$	Efficacy of rGM-CSF treatment	See text	
$\varepsilon_c$	Efficacy of clindamycin treatment	See text	
$\varepsilon_a$	Bacterial death rate from ampicillin treatment	11.35	day^−1^
$\varepsilon_i$	Bacterial death rate from additional immune responses under clindamycin treatment	3.0	day^−1^

### Initial dose threshold

Equations (5–9) were previously used to derive an initial dose threshold that describes the relationship between bacterial dose/load and AM depletion [38]. This threshold is defined by Eq. (10), which is the unstable steady state solution ($T^*$, $I_1^*$, $I_2^*$, $V^*$, $P^*$) = (0,0,0,0,$P^*$) when $\hat{\phi }>0$. $P^*$ satisfies $P^3+BP^2+CP+D=0$. This state separates the two stable steady states (0,0,0,0,0) and (0,0,0,0,$K_P$) and is constant when virus-induced AM depletion is absent (i.e., when $\hat{\phi }=0$) and dynamic when virus-induced AM depletion is present (i.e., when $\hat{\phi }>0$). The threshold, shown in Fig. 2b, dictates whether bacteria exhibit a growth phenotype (to stable state $P^*$ = $K_P$) or a clearance phenotype (to stable state $P^*$ = 0). That is, bacterial loads decrease for dose-depletion pairings below the threshold and increase for dose-depletion pairings above the threshold. Additional details can be found in Ref. [38] along with the experimental validation of these dynamics.

10 $$\begin{aligned} P^*&=-\frac{1}{2}(M_1+M_2)- \frac{\sqrt{3}}{2}(M_1-M_2)i-\frac{B}{3} \end{aligned}$$

where

$$\begin{aligned} M_{1,2}&=\root 3 \of {-\frac{q}{2}\pm \sqrt{\frac{u^3}{27}+\frac{q^2}{4}}}\\ u&=C-\frac{B^2}{3}\\ q&=D+\frac{2B^3-9BC}{27}\\ B&=-K_P\\ C&=n^2M_A\\ D&=n^2M_AK_P\left( \frac{\gamma_{M_A}M_A}{r}(1-\hat{\phi })-1\right) \end{aligned}$$

For the parameter values in Table 1, $P^*$ is real if $\frac{\gamma_{M_A}M_A}{r}(1-\hat{\phi })>1$ (i.e., $D>0$). However, $P^*$ is complex when $\frac{\gamma_{M_A}M_A}{r}(1-\hat{\phi })<1$ (i.e., $D<0$). The point where $P^*$ switches from being a real root to a complex root with real part less than 1 is found by solving $D=0$ for $\hat{\phi }$ or *r*, which gives the critical values

11 $$\begin{aligned} \hat{\phi }_{crit}&=1-\frac{r}{\gamma_{M_A}M_A} \end{aligned}$$

12 $$\begin{aligned} r_{crit}&=(1-\hat{\phi })\gamma_{M_A}M_A \end{aligned}$$

**Fig. 1:**
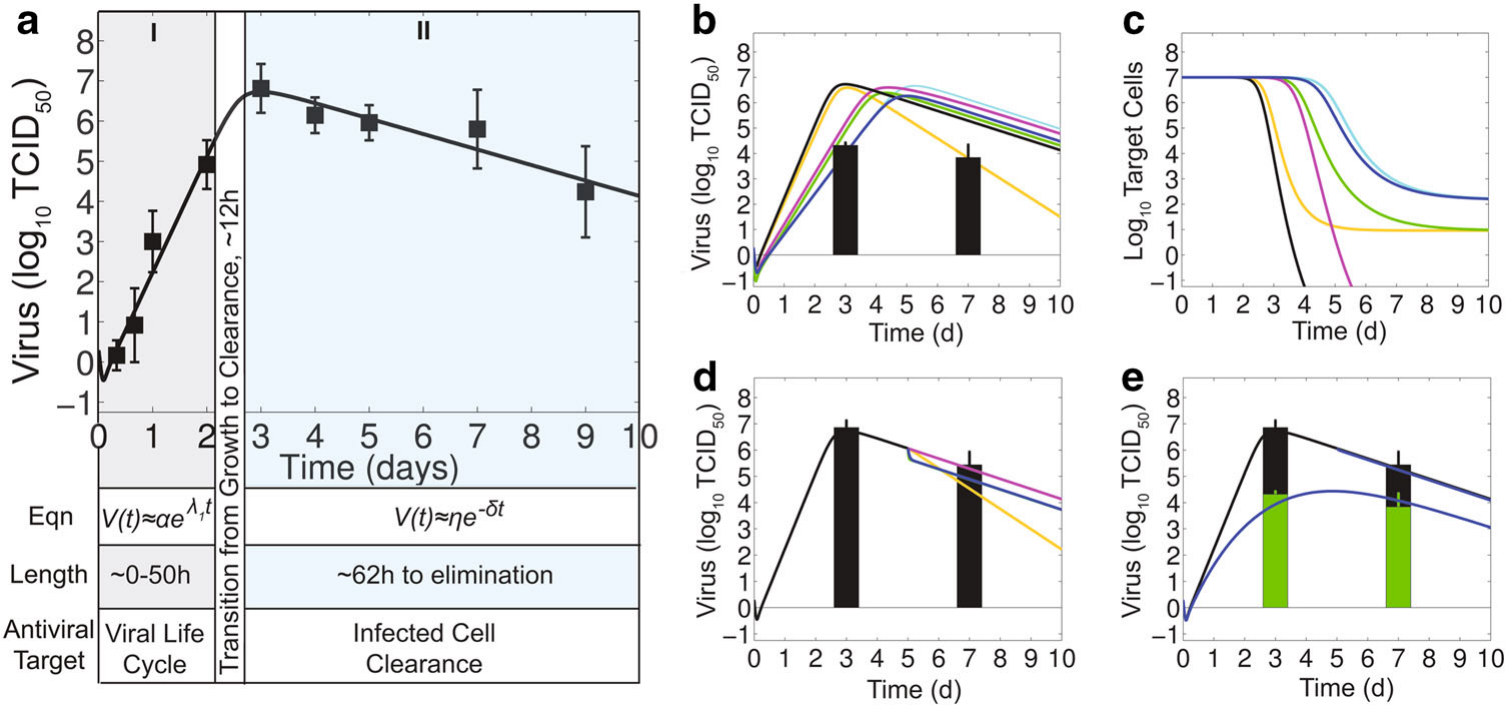
Breakdown of viral kinetics and effects of antiviral therapy. **a** Fit (*black line*) of Eqs. (1–4) to viral titers in the lungs of mice infected with 100 TCID$_{50}$ PR8 (*black squares*) [41]. The equations and time scale that characterize the phases of exponential growth (*shaded in gray*), the transition from growth to clearance (*shaded in white*), and the exponential decay (*shaded in blue*) [40] are shown along with the most effective antiviral target. **b**–**d** Simulation of Eqs. (1–4) against viral load data under NAI therapy given prophylactically (*Panels b–c*) or at 5 days pii (*Panel d*) [21] for no therapy (*black line*) and for different antiviral targets [virus infection ($\beta$, *cyan line*), eclipse phase (*k*, *magenta line*), virus production (*p*, *blue line*), virus clearance (*c*, *green line*), or infected cell clearance ($\delta$, *orange line*)] with efficacy $\varepsilon_v$ = 60 %. **e** Simulation of Eqs. (1–4) against viral load data under NAI therapy given prophylactically (*green bars*) or at 5 days pii (*black bars*) [21] assuming that NAIs inhibit virus production ($p(1-\varepsilon_v)$) with efficacy $\varepsilon_v$ = 10 % and protect target cells from being infected (Eq. (14)) at rate $\varepsilon_t$ = 0.68 day$^{-1}$. The parameters values used for all simulations are provided in Table 1 (Color figure online)

**Fig. 2:**
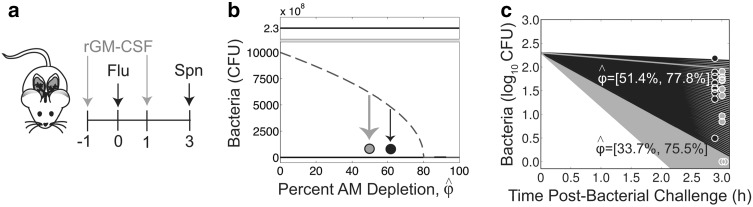
Effect of rGM-CSF therapy. **a** Therapeutic schedule used to evaluate rGM-CSF therapy in mice infected with PR8 (“Flu”) followed by 200 CFU A66.1 (“Spn”) [8]. **b** Simulation of Eq. (10) with the parameters in Table 1 and various values of $\hat{\phi }$. Dose-AM depletion pairing and distance from the threshold are illustrated for no therapy (*black*) and rGM-CSF therapy (*green*). **c** Simulation of Eqs. (5–9) against bacterial load data under no therapy (*black*) or rGM-CSF therapy (*green*). The parameters used are those in Table 1 with the indicated range of AM depletion ($\hat{\phi }$) (Color figure online)

### Therapeutic benefit

The area under the curve (AUC) is used to quantify the benefit of administering a particular drug. To estimate the therapeutic benefit, the pathogen load was estimated at discrete time points by the numerical solution to Eqs. (5–9) or to Eq. (10) and the trapezoidal rule is used to estimate the AUC.

### Data

To investigate infection kinetics under therapy, data from three published studies were used [8, 12, 21]. These studies use the same experimental model system that was employed to parameterize the viral infection model (Eqs. (1–4)) and the coinfection model (Eqs. (5–9)) [41, 43]. The data from each study was digitized using PlotDigitizer [31].

#### Data under antiviral therapy

The viral titer data used to investigate the dynamics under antiviral therapy was taken from Ref. [21]. In this study, groups of 6–8 weeks old female BALB/cJ mice (Jackson Laboratory, Bar Harbor, ME) were lightly anesthetized with 2.5 % inhaled isoflurane and infected intranasally with 50 TCID$_{50}$ PR8 in 100 ul. Mice were then mock-treated with PBS or given a neuraminidase inhibitor (NAI) (oseltamivir, 5 mg/kg) twice daily by oral gavage for 5 days beginning 4 h before infection (prophylaxis) or 5 days pii (late administration). Mice were euthanized by CO$_2$ inhalation at 3 days pii or 7 days pii and the viral titers were enumerated.

#### Data under GM-CSF therapy

The bacterial titer used to investigate the dynamics under GM-CSF therapy was taken from Ref. [8]. In this study, groups of 6–8 weeks old female BALB/cJ mice (Jackson Laboratory, Bar Harbor, ME) were given 25 μg recombinant granuloctye macrophage colony stimulating factor (rGM-CSF) intranasally in 100 μl 1 day before and 1 day after infection with PR8 at a dose of 25 TCID$_{50}$ in 100 ul. Groups of mice were then mock-infected with PBS or infected with 200 CFU pneumococcus A66.1 at 3 days pii. For all infections, mice were lightly anesthetized with 2.5 % inhaled isoflurane. After euthanasia by CO$_2$ inhalation at 3 h post-bacterial infection (pbi), bronchoalveolar lavage fluid (BALF) was collected and the bacterial titers were enumerated.

#### Data under antibiotic therapy

The bacterial titer data used to investigate the dynamics under antibiotic therapy was taken from Ref. [12]. In this study, groups of 6–8 weeks old female BALB/cJ mice (Jackson Laboratory, Bar Harbor, ME) were lightly anesthetized with 2.5 % inhaled isoflurane and infected with 37 TCID$_{50}$ PR8 in 100 μl then with 200 CFU pneumococcus A66.1 in 100 μl at 7 days pii. Bioluminescent imaging was used to monitor the development of pneumonia. At the onset of pneumonia, mice were mock-treated with PBS or treated with ampicillin (100 mg/kg) or clindamycin (15 mg/kg) administered by intraperitoneal injection twice daily. The bacterial titers were enumerated by bioluminescent imaging in live mice at 0, 12, and 24 h after treatment initiation. The data are reported as relative light units (RLU) per minute. To explore these data with a model that has parameters with units in CFU, a log-log correlation between CFU and RLU (Fig. S3) is used and defined by Eq. (13).

13 $$\begin{aligned} \log_{10}\mathrm {(RLU)}=0.448\log_{10}\mathrm {(CFU)}+2.1068 \end{aligned}$$

## Results

### Dynamics under antiviral therapy

Antivirals reduce the viral load and, in turn, lessen the disease severity. This is sufficient to reduce the morbidity and mortality caused by SBIs [18, 21]. Prophylaxis with NAIs can reduce viral titers by 2.5–3.0 $\log_{10}$ TCID$_{50}$ and SBI mortality by 50 %, whereas late administration (beginning at 5 days pii) results in $0.8 \log_{10}$ TCID$_{50}$ lower viral loads and 33 % less SBI-associated mortality (see 'Methods' section) [21].

The differential efficacy of early versus late administration has been explained by using Eqs. (1–4), where approximate solutions of the model define the contribution of each infection process (e.g., virus infection, production, clearance, etc.) [40]. In brief, the model solution during the growth phase of the virus (first 2 days pii) indicates that the processes dominating the kinetics are those that are targeted by antivirals, i.e., $V(t)=\alpha e^{\lambda t}$, where $\lambda$ is a combination of all model parameters. However, the slower rate of virus growth after this time suggests that the infection processes are changing and that this is the point where antivirals that target the viral life cycle begin to lose their efficacy. The later stages of infection (>3 days pii) are dominated by a single process, i.e., infected cell clearance ($V(t)=\eta e^{-\delta t}$). With a single parameter controlling the rate of virus load decay, an antiviral that targets this process may be more efficacious. These dynamics are summarized in Fig. 1a.

To further illustrate the time-dependent changes in antiviral efficacy, Eqs. (1–4) were simulated assuming that NAIs inhibit the rate of virus production (*p*) with efficacy $\varepsilon_v$ (i.e., $p(1-\varepsilon_v)$). When therapy is initiated at 0d pii (prophylaxis), setting $\varepsilon_v$=60 % matches the viral titer data at 3 days pii but fails to capture the lower titer at 7 days pii (Fig. 1b). When therapy is initiated at 5 days pii (late administration), there is little change in the viral titers and an efficacy of $\varepsilon_v$=60 % overestimates the decline (Fig. 1d). Using the same efficacy ($\varepsilon_v$=60 %) to investigate therapeutically targeting other infection processes suggests that there is little difference between targeting virus production (*p*) and infection ($\beta$), that increased efficacy is needed for therapies directed against virus replication (*k*) or clearance (*c*), and that a therapy designed to increase the rate of infected cell clearance ($\delta$) could result in faster clearance (Fig. 1b–d). Perturbing any of these processes with the exception of the eclipse phase (*k*) also resulted in fewer cells becoming infected if the antiviral is given prophylactically (Fig. 1c). However, there is no effect on target cells with late administration due to these cells being depleted by 5 days pii (not shown).

The model in Eqs. (1–4) excludes specific host responses, which may be altered when virus production (*p*) is inhibited by NAIs. Increasing the rate of virus clearance (*c*) in addition to the rate of virus production could not reproduce the data. Increasing the rate of infected cell clearance ($\delta$) could capture the dynamics under NAI prophylaxis but not under late administration (not shown). Alternatively, innate immune responses (e.g., interferons) or other host factors may remove target cells (*T*) from the susceptible pool altogether, which is modeled with the Eq. (14).

14 $$\begin{aligned} \frac{dT}{dt}&=-\beta TV -\varepsilon_tT, \end{aligned}$$

where $\varepsilon_t$ is the rate that these cells become protected. Including this effect in Eqs. (1–4) in addition to the inhibition of virus production ($p(1-\varepsilon_v)$) and setting $\varepsilon_v$ = 10 % and $\varepsilon_t$ = 0.68 d$^{-1}$ can simultaneously reproduce the data under NAI therapy at both 0 days pii and 5 days pii (Fig. 1e).

### Dynamics under immunotherapy

Because AM depletion drives pneumococcal establishment during influenza virus infection [8, 38, 43], restoring the AM population or preventing the depletion reduces bacterial burden [8] and may be able to prevent pneumococcal invasion altogether. Mice treated with rGM-CSF 1 day before and 1 day after infection with PR8 (Fig. 2a, see 'Methods' section) exhibited an average increase of $\sim$20 % in the AM population 2 days after the end of treatment (at 3 days pii) [8]. This correlated to an average decrease of $\sim$16 % in the bacterial loads within 3 h pbi with 2 out of 10 mice achieving resolution within 3 h pbi, and a 60 % reduction in pneumonia.

In the absence of treatment, AMs are depleted by 64.6 ± 13.2 %, on average, at 3 days pii [8]. This correlates to a threshold value (Eq. (10)) of $4.4\pm 2.1\,\times\, 10^3$ CFU [38]. Because the inoculating dose was 200 CFU, which is well below this threshold value (Fig. 2b), bacteria clear rapidly and $\sim$24 % of the inoculum remains at 3 h pbi (Fig. 2c) [8]. Using the percentage of AM depletion as the value of $\hat{\phi }$, Eqs. (5–9) were simulated across the data range ($\hat{\phi }=51.4-77.8$ % AM depletion) and found to capture the empirical measurements accurately (Fig. 2c).

Under rGM-CSF therapy, AM depletion is reduced by $\varepsilon_g=18.7\pm 15.7$ % [8] (i.e., $\hat{\phi }(1-\varepsilon_g$), where $\varepsilon_g$ is the efficacy of rGM-CSF), which moves the position on the dose-depletion curve to a location further away from the threshold (Fig. 2b). This suggests that the bacteria will clear at a faster rate for the same dose [38]. Indeed, only $\sim$8 % of the inoculum remains at 3h pbi compared to 24 % in the absence of treatment. These dynamics are accurately predicted by Eqs. (5–9) with values of $\hat{\phi }$ between 33.7 % and $75.5\,\%$ (Fig. 2c). The additional clearance potential (the additional area of green in Fig. 2c) corresponds to a 33 % decrease in the AUC, which is used to quantify the therapeutic benefit. The reduction in AM depletion is sufficient to allow for resolution in some mice, which the model suggests may have occurred as early as 2h pbi as indicated by the lower green line in Fig. 2c.

**Fig. 3:**
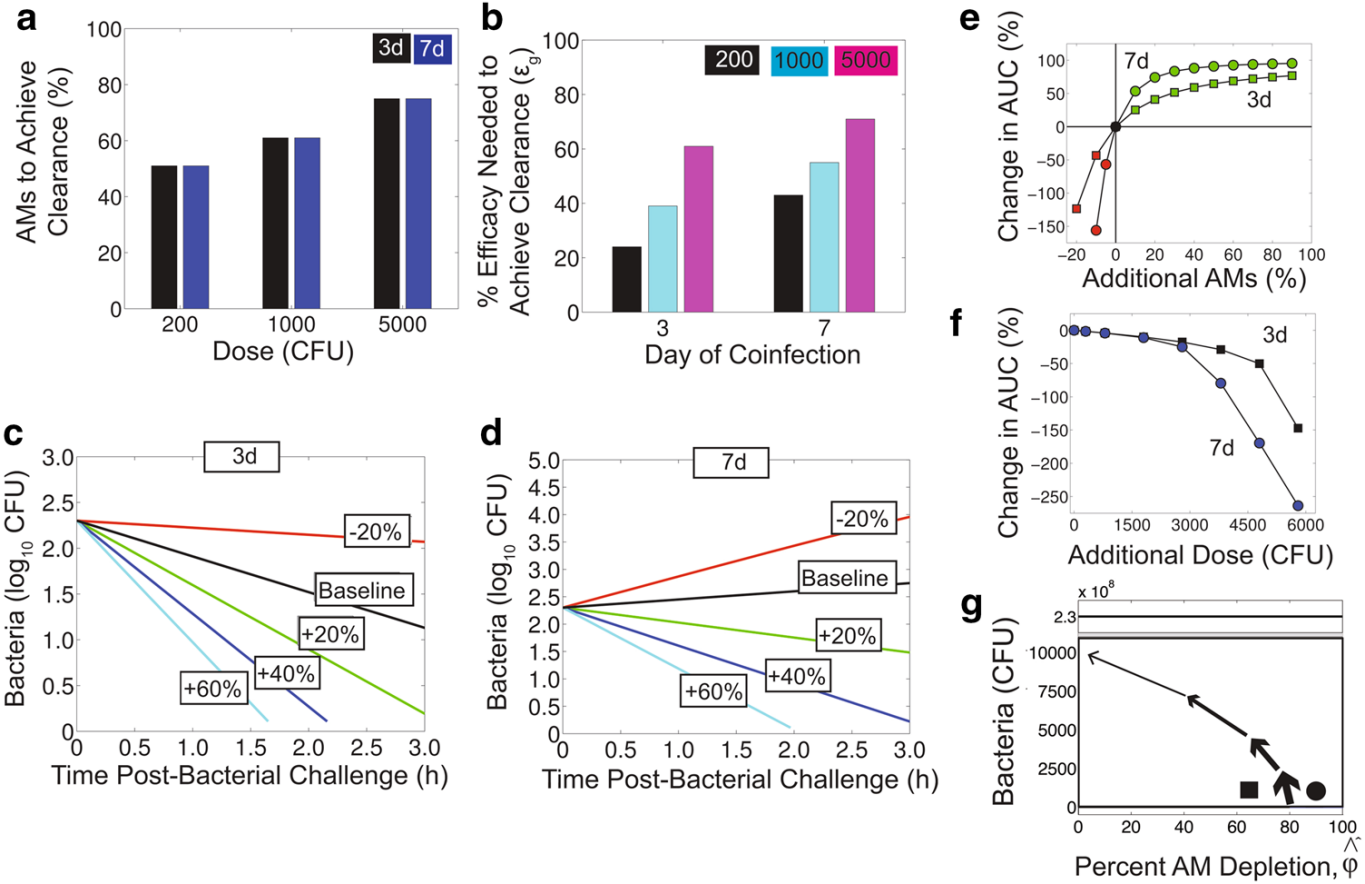
Differential therapeutic benefit of decreasing AM depletion. **a** Minimum percentage of AMs ($1-\hat{\phi }$) needed to achieve resolution ($P=0 \log_{10}$ CFU) by 3 h pbi for coinfections at 3 days pii (*black*) or 7 days pii (*blue*) and for bacterial doses of 200 CFU, 1000 CFU, or 5000 CFU. **b** Percent efficacy ($\varepsilon_g$) needed to achieve resolution by 3 h pbi for a coinfection at 3 days pii or 7 days pii and for a bacterial dose of 200 CFU (*black*), 1000 CFU (*cyan*), or 5000 CFU (*magenta*). **c**–**d** Simulation of Eqs. (5–9) for different percentage increases in AMs calculated from baseline for a coinfection at 3 days pii (*Panel c*) or 7 days pii (*Panel d*). **e**–**f** Calculated therapeutic benefit (change in AUC) for different percentage increases in the AM population ($\hat{\phi }$) (*Panel e*) or for different dose increases (*Panel f*) for a coinfection at 3 days pii (*squares*) or 7 days pii (*circles*). *Green* indicates a positive therapeutic benefit and *red* indicates a negative therapeutic benefit (*Panel e*). **g** Schematic showing how the slope of the threshold and, thus, the therapeutic benefit increases more rapidly for higher degrees of AM depletion. Baseline values of AM depletion at 3 days pii (*square*) and 7 days pii (*circle*) are shown for a dose of 200 CFU. Unless otherwise noted, the numerical solution to Eqs. (5–9) with the parameters in Table 1 was used. Baseline is $\hat{\phi }=64.6$ % for a coinfection at 3 days pii and $\hat{\phi }=87$ % for a coinfection at 7 days pii (Color figure online)

Although two mice resolved the infection and presumably had lower levels of AM depletion, the other eight mice had bacteria remaining at 3 h pbi. These individuals may have been among the 40 % that progressed to pneumonia because bacterial growth can be restored if clearance is incomplete within 3–4 h pbi [38]. Using the numerical solution to Eqs. (5–9) to find the minimum number of AMs ($1-\hat{\phi }$) needed to achieve resolution ($\log_{10}(P)=0$) by 3 h pbi indicated that at least 51 % of the AM population is required to clear a dose of 200 CFU (Fig. 3a). This corresponds to an efficacy of $\varepsilon_g=24$ % (i.e., $\hat{\phi }=64.6$ $\rightarrow 49$ %) (Fig. 3b). Interestingly, the critical number of AMs needed to resolve the infection is conserved across coinfection timings (3 vs 7 days pii) (Fig. 3a), but the percentage of AM replenishment required is larger at 7 days pii because the baseline value of AM depletion is greater at this time ($\hat{\phi }=87$ %) than at 3 days pii ($\hat{\phi }=64.6$ %) (Fig. 3b). This can also be seen by simulating the coinfection model (Eqs. (5–9)) for other percentage increases in the AM population (Fig. 3c, d). Calculating the therapeutic benefit (AUC) for various increases in the AM population for a coinfection at 3 days pii or 7 days pii suggests that the greatest benefit occurs when AM depletion is high (large $\hat{\phi }$, more severe infection) and, thus, later in the infection (7 vs 3 days pii) (Fig. 3e). Similarly, the therapeutic benefit is greater if the infection is more severe as a result of a high inoculating dose (Fig. 3f). The more robust response to therapy is due to the greater slope of the threshold with large $\hat{\phi }$ (high degree of AM depletion), as illustrated in Fig. 3g.

### Dynamics under antibiotic therapy

The bacterial burden, which can be reduced directly by antibiotics, contributes to pathogenicity of SBIs during influenza virus infections. Treating coinfected mice with a cell wall active agent (ampicillin) or a protein synthesis inhibitor (clindamycin) at the onset of pneumonia (see 'Methods' section) showed that ampicillin could reduce bacterial titers considerably, whereas clindamycin had a limited ability to reduce titers but lessened the disease severity by reducing inflammation [12]. This correlated to a 50 % increase in survival in the ampicillin treated mice and an 80 % increase in survival in the clindamycin treated mice.

To examine the effect of antibiotics on coinfection kinetics, I assume that pneumococci are killed at rate $\varepsilon_a$ d$^{-1}$ by ampicillin, which directly lyses bacteria, and that the replication rate (*r*) is reduced with efficacy $\varepsilon_c$ by clindamycin, which inhibits replication. Adding these effects to Eq. (9) yields

15 $$\begin{aligned} \frac{dP}{dt}&=(1-\varepsilon_c)rP\left( 1-\frac{P}{K_P}\right) -\gamma_{M_A} f(P,M_A)PM_A(1-\hat{\phi }(V))-\varepsilon_a P. \end{aligned}$$

To match the model output to the data, it was first necessary to estimate the time at which therapy was initiated because the authors of the study reported only that therapy began at the onset of pneumonia as visualized by bioluminescent imaging. A reasonable value for the start time was obtained by simulating Eqs. (5–9) in the absence of antibiotics ($\varepsilon_a=0$, $\varepsilon_c=0$) until the numerical solution [adjusted to RLU with Eq. (13)] matched the first data point. This resulted in a start time for mock treated mice of 10 h pbi, with the respective start times for the clindamycin and ampicillin treated mice being 48 min and 91 min later. With six mice per group, these times correlate to a process time of 7–8 min per mouse, which is a reasonable length of time to identify the onset of pneumonia through bioluminescent imaging and administer treatment.

**Fig. 4:**
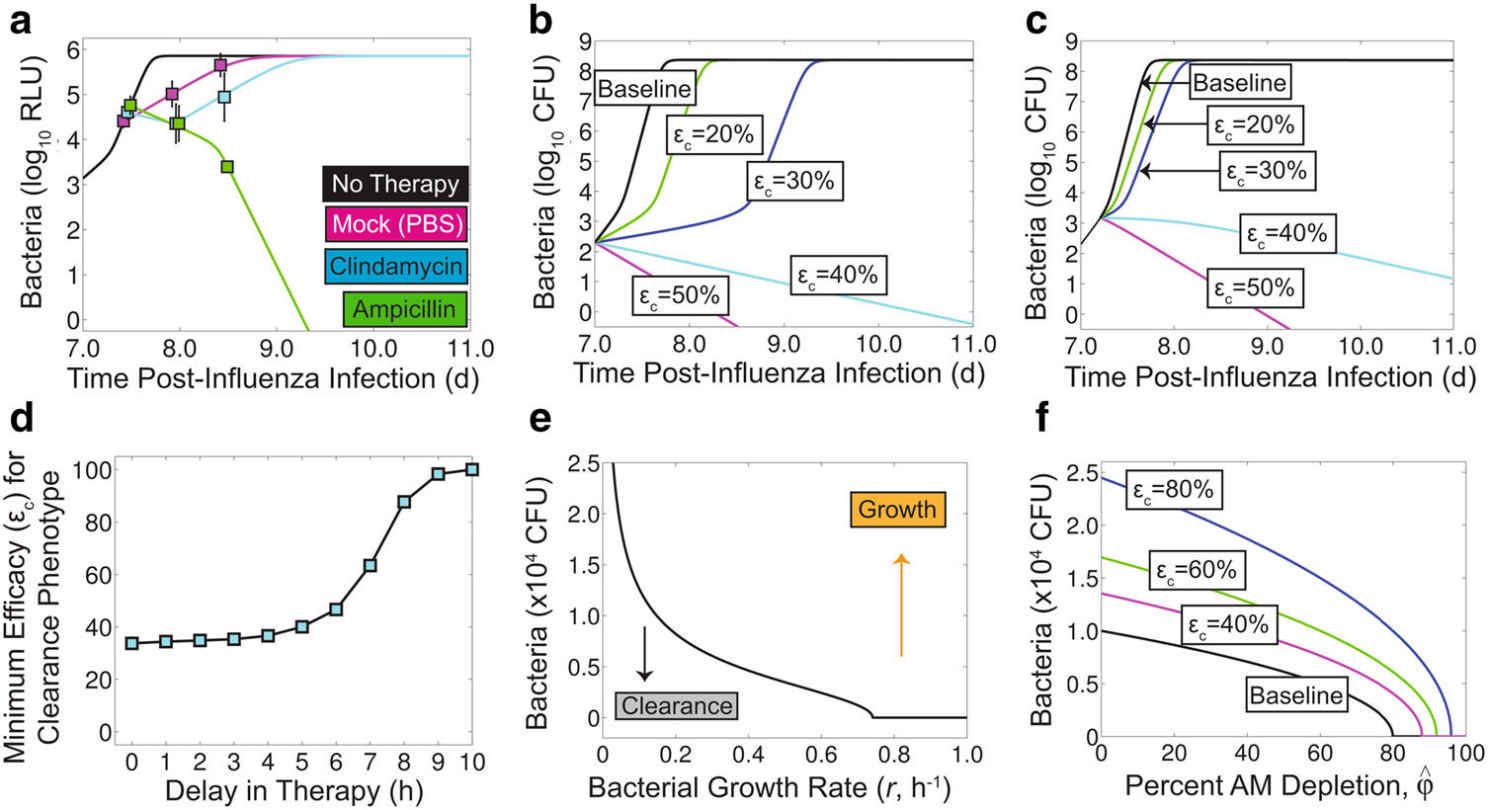
Effect of antibiotic therapy and potential for combination therapy. **a** Simulation of Eqs. (5–8) and (15) against bacterial load data (obtained by bioluminescent imaging, RLU) under mock therapy (*magenta*) or antibiotics [ampicillin (*green*) or clindamycin (*cyan*)] [12]. The parameters used are those in Table 1 with $\varepsilon_{a,c,i}=0$ (for no therapy and mock therapy), *r* = 6.5 d^−1^ (for mock (PBS) and antibiotic therapy), $\varepsilon_a=11.35 \; \text d^{-1}$ (for ampicillin), and $\varepsilon_c = 1$ and $\varepsilon_i = 3\; \text d^{-1}$ until $\sim$8 days pii and $\varepsilon_{c,i}=0$ thereafter (for clindamycin). The model output was adjusted to RLU with Eq. (13). **b**–**c** Simulation of Eqs. (5–8) and (15) for various values of antibiotic efficacy ($\varepsilon_c$) for prophylactic treatment (beginning at 0d pbi, *Panel b*) or delayed treatment (beginning at 5 h pbi, *Panel b*). **d** Minimum efficacy ($\varepsilon_c$) needed to achieve a clearance phenotype found by simulating Eqs. (5–8) and (15) for treatment beginning at various times pbi. **e**–**f** Simulation of the threshold solution (Eq. (10)) with various values of the bacterial growth rate (*r*) alone (*Panel e*) or in addition to the degree of AM depletion ($\hat{\phi }$) (*Panel f*) (Color figure online)

Simulating Eq. (15) together with Eqs. (5–8) beginning at the times indicated above suggests that the mock treated mice may have also had altered bacterial kinetics (Fig. 4a). For the mock treated group ($\varepsilon_a=0$, $\varepsilon_c=0$), the model could reproduce the data if the replication rate (*r*) was reduced to 6.5 d$^{-1}$. When this value was used to simulate antibiotic treatment, ampicillin could eliminate bacteria at a rate of $\varepsilon_a=11.35$ d$^{-1}$.

For clindamycin therapy, setting $\varepsilon_c=1$ (i.e., 100 % efficacy) inhibited growth but did not result in decreased bacterial loads (not shown). Incorporating additional clearance at a rate of $\varepsilon_i=3$ d$^{-1}$ [i.e., replacing $\varepsilon_a$ with $\varepsilon_i$ in Eq. (15)] could produce the decline seen in the data during the first 12 h after therapy. However, the data at 24 h post-treatment showed a rebound in the bacterial titers, and, thus, the model deviates from the data after 12 h post-treatment when antibiotic effects are included (not shown). Removing the entire effect of antibiotics and the additional immune-mediated clearance (i.e., setting $\varepsilon_{c,i}=0$) at 12 h post-treatment could restore the model accuracy for later time points. The growth in bacterial titers at this point occurs at the same rate as in the mock treated mice, suggesting a complete loss of efficacy of the antibiotic.

While clindamycin works to eliminate bacteria through non-lytic mechanisms, other antimicrobial agents that target pathogen replication could also be efficacious. In addition, earlier administration of the drug should be beneficial. To quantify how much a drug would need to reduce the growth rate in order to be effective, Eqs. (5–8) and (15) were simulated for various values of $\varepsilon_c$ assuming that therapy begins at 0d pbi (prophylaxis) or at 5 h pbi (delayed). The model solution indicated that prophylactic administration would require increasing the doubling time from 37 min to 56 min ($\varepsilon_c=33.7\,\%$) to enable AMs to control the infection and achieve an immediate clearance phenotype (Fig. 4b). Delaying treatment to 5 h increased this doubling time to 61.5 min ($\varepsilon_c=40\,\%$; Fig. 4c). If treatment is delayed even further, the minimum efficacy required to result in a clearance phenotype increases rapidly and 100 % efficacy is required at 10 h pbi (Fig. 4d), which is consistent with the results above that indicated the efficacy ($\varepsilon_c$) of clindamycin therapy initiated after 10 h pbi was 100 %.

The nonlinearity of the required efficacy in Fig. 4d and the dynamics in Fig. 4b–c illustrate that the replication rate (*r*) is a bifurcation parameter, similar to the AM depletion parameter ($\hat{\phi }$). That is, differential dynamics occur depending on the value of *r*. Indeed, the threshold solution (Eq. (10)) is dependent on two parameters other than $\hat{\phi }$, the rates of bacterial replication (*r*) and clearance ($\gamma_{M_A}$) (see 'Methods' section) [38]. Because $\gamma_{M_A}$ would be difficult to therapeutically manipulate, the remaining analyses focus on *r*. Plotting the solution to Eq. (10) for various values of *r* while keeping all other parameters fixed to the values in Table 1 illustrates the response to inhibiting the growth rate (Fig. 4e). That is, the increasing area under the threshold for decreasing values of *r* indicates a greater opportunity for bacterial clearance. The critical value (Eq. (12)) where clearance potential is gained is $r_{crit}=0.74$ h$^{-1}$, which corresponds to a doubling time of 56.2 min.

### Potential for combination therapy

Thus far, I have examined how different therapeutic approaches can alter two different parameters of the coinfection model that drive the dynamics [i.e., the degree of AM depletion ($\hat{\phi }$) and the bacterial growth rate (*r*)]. Because the coinfection dynamics are sensitive to changes in both parameters, it is possible that they can be altered simultaneously through combination therapy with, for example, an antibiotic (e.g, clindamycin) and immunotherapy (e.g., rGM-CSF) or an antiviral (e.g., NAI), if reducing the viral load also reduces AM loss. Plotting the threshold solution (Eq. (10)) for different values of $\hat{\phi }$ and *r* shows how the threshold increases as the rate of bacterial replication is reduced (i.e., increasing the efficacy, $\varepsilon_c$; Fig. 4f). The larger distance below the threshold, which correlates to the rate of bacterial clearance, with increasing antibiotic efficacy ($\varepsilon_c$) suggests a significant gain in clearance potential with combination therapy. If the AUC of the threshold is used as a measure of therapeutic potential, an antibiotic efficacy of 40 %, 60 % or 80 % increases the chances of successful treatment with immunotherapy and/or antivirals by 49 %, 95 %, or 194 %, respectively.

## Discussion

Given the severity of influenza virus infections and influenza-associated secondary bacterial infections, effectively preventing both infections is crucial. The limited protection and availability of vaccines, together with the inadequacies of antimicrobial agents, make treating SBIs challenging. Although suboptimal efficacy may be unavoidable to some extent, a detailed understanding of how infection processes change over time and the feedbacks between various pathogen and host factors aids our ability to develop new therapeutic strategies and/or targets that effectively abrogate influenza infections and SBIs.

**Fig. 5:**
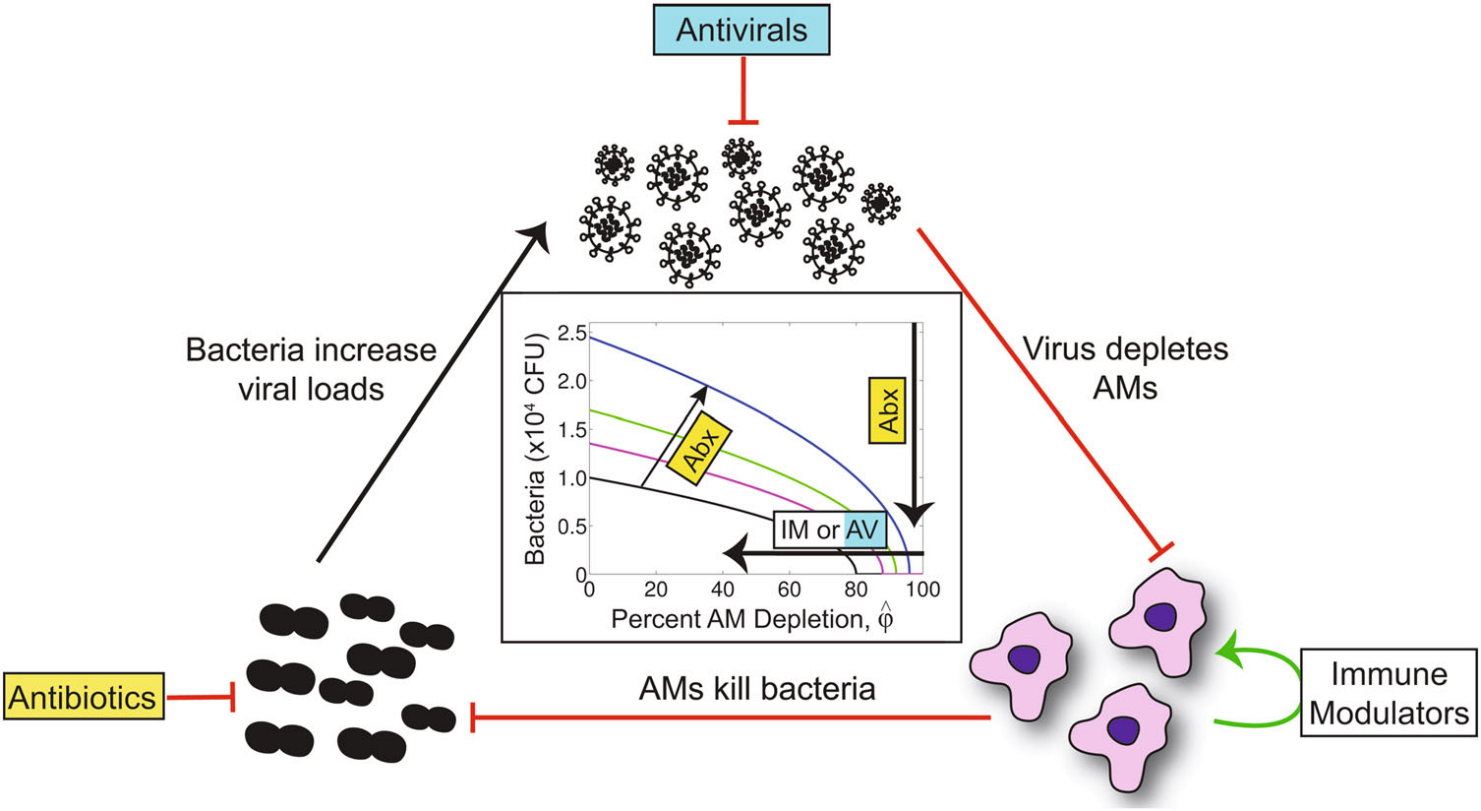
Summary of therapeutic strategies to combat SBIs during influenza. Schematic of the regulating mechanism driving SBIs during influenza virus infections and various therapeutic strategies targeted at each process. Influenza virus infection results in the depletion of alveolar macrophages (AMs), which in turn allows for bacteria to invade and grow. This bacterial growth then increases the viral load. Antiviral therapy (AV) can reduce virus growth, which may in turn decrease AM depletion. AM depletion can be reduced by immunotherapy (IM), which improves bacterial clearance. Antibiotics (Abx) can reduce the bacterial loads and/or the bacterial growth rate, which may reduce the post-bacterial viral load rebound. The figure in the center shows the relationship between AM depletion (x-axis), bacteria load (y-axis), and bacterial growth rate (*colored lines*), as defined by Eq. (10). Values above/below the threshold lines support growth/clearance phenotypes. Also depicted are the ways in which each therapy can be used alone or in combination (i.e., by using Abx to slow bacterial growth (from the *black line* to the *blue line*) or to reduce bacterial loads, and by using IM or AV to reduce AM loss) (Color figure online)

By utilizing kinetic models describing influenza virus infection [2, 41] and bacterial coinfection [43] and exploiting the tight correlation between two factors (i.e., bacterial dose/load and AM depletion) that regulate bacterial acquisition and initial bacterial titer trajectories [8, 38, 43], the analysis here shows how infection kinetics change when different processes are perturbed with antimicrobial agents. Given that virus infection $\rightarrow$ AM loss $\rightarrow$ reduced bacterial clearance $\rightarrow$ increased viral load, therapeutically targeting these processes should have similar effects (Fig. 5). However, the nonlinearity of the relationship between AM depletion, the bacterial load/dose, and the bacterial growth rate (Figs. 4f, 5) illustrates that the extent to which pathogen loads can be therapeutically reduced is dependent on the time of administration (i.e., location on the threshold axes) and the mechanism of action of the drug (Figs. 3, 4, 5). In addition, the steeper slope of the threshold for high values of AM depletion (Fig. 3g) highlights the faster response to therapies that decrease the depletion when the infection is more severe, but this is complicated by a greater therapeutic efficacy needed to resolve the infection (Fig. 3). The response to therapy will be increased further when bacterial growth is also inhibited (i.e., during combination therapy) because the slope of the threshold is steeper (Fig. 4f). The additional area under the threshold with combination therapy also suggests that it may be possible to decrease the amount of drug used and/or the length of therapy. In contrast, manipulating only the growth rate would require a higher efficacy than manipulating the AM population because of the slower rate of change of the curve in Fig. 4e.

Although the mechanism resulting in AM loss during influenza is unknown, viral loads may be directly related to AM depletion [38]. Data obtained by manipulating the dose-depletion combination suggested that when bacterial loads decline in the first 4 h pbi (i.e., dose-depletion pairing below the threshold), but do not clear, bacteria can overcome AMs and switch to a growth phenotype [38]. When this occurs, it results in a large degree of heterogeneity in bacterial loads at 24 h pbi, whereas little heterogeneity results from dose-depletion pairings above the threshold [38]. This observation provided insight into the coinfection dynamics with the PR8-PB1-F2(1918) virus, for which bacterial titers diverged by 24 h pbi [43]. In these mice, viral titers were also lower at the onset of the coinfection [41, 43], which suggested a dose-depletion pairing below the threshold. Although it is unknown if there was less AM depletion with this virus, the correlation led to the hypothesis that viral titers are linked to the depletion of these cells. This may help explain why even small reductions in the viral load as a result of antiviral treatment can lead to substantial reductions in SBI morbidity and mortality [21]. It is also possible that reducing viral loads with antiviral treatment has additional effects on host immune responses or other host factors (e.g., as in Fig. 1e) that are beneficial in decreasing the incidence and pathogenicity of SBIs. With several anti-influenza drugs not yet licensed for use in the U.S. or under development (reviewed in [11]), it will be important to test their effect on immune components and their efficacy in animal coinfection models.

Treatment with antiviral agents may prevent the detrimental effects on AMs during influenza virus infection, but the cell population can also be restored by immunotherapy with agents like rGM-CSF [8]. Even with a short treatment regimen that resulted in $\sim$20 % efficacy, bacterial clearance for a low dose infection could be improved, generating a 33 % therapeutic benefit. However, the results presented here suggest that more severe infections, such as those initiated by larger doses, would require significantly greater efficacy (Fig. 3a, b). It is unclear if another treatment schedule with rGM-CSF or an alternate drug could improve these figures. Because rGM-CSF therapy has some drawbacks, such as increasing inflammation (reviewed in [10]), that may inhibit its use during influenza-associated diseases, developing other therapies that increase the AM population is necessary.

Reducing the pathogen load is the goal of many therapeutics, including antivirals and antibiotics, but the pathogen titers do not always correlate with disease. Further, reducing inflammation directly or through reduction of the pathogen burden often leads to a better outcome. Protein synthesis inhibitors (e.g., clindamycin), which slow bacterial replication in addition to having anti-inflammatory effects [12, 13], are one example of such a treatment. However, decreasing the inflammatory response leads to a rebound in bacterial loads [12] (Fig. 4a), which has been attributed to a lower neutrophil influx into the lungs mediated by Toll-like receptor 2 (TLR-2) [13]. This may explain why it was necessary to remove the effect of antibiotics ($\varepsilon_c$) and the additional immune response ($\varepsilon_i$) in Eq. (15) shortly after therapy initiation in order to match the data (Fig. 4a). Although neutrophil dynamics are currently excluded from the model, $\varepsilon_i$ reflects bacterial phagocytosis by these cells. An understanding of the relative effects of neutrophils on pathogen kinetics and inflammation/disease may aid the design of new therapeutic approaches, particularly given that these cells undergo influenza-induced apoptosis and become dysfunctional during SBIs [5, 6, 14, 23, 30, 36] and that TLR-2 antagonists can protect against SBIs [26].

Kinetic models provide a robust means of evaluating how infection kinetics change when different processes are perturbed by therapeutics. These models yield important information about the feasibility of attaining a particular outcome (e.g., clearance within a distinct time frame), the off-target effects of a drug (e.g., on immune responses), and the time-scale on which a drug is most effective. In addition, establishing how different mechanisms are related pinpoints strategies that can simultaneously alter each pathway and provides insight into the impact of using multiple therapies. Determining how other pathogen and host factors work together will undoubtedly identify new therapies for these diseases.

## Electronic supplementary material

Below is the link to the electronic supplementary material.

Supplementary material 1 (pdf 8392 KB)

## References

[1] Aoki F, Macleod M, Paggiaro P, Carewicz O, El Sawy A, Wat C, Griffiths M, Waalberg E, Ward P, IMPACT Study Group, et al (2003) Early administration of oral oseltamivir increases the benefits of influenza treatment. J Antimicrob Chemother 51(1):123–129 10.1093/jac/dkg007 12493796

[2] Baccam P, Beauchemin C, Macken C, Hayden F, Perelson A (2006). Kinetics of influenza A virus infection in humans. J Virol.

[3] Brundage J, (2006) Interactions between influenza and bacterial respiratory pathogens: Implications for pandemic preparedness. Lancet Infect Dis 6(5):303–312 10.1016/S1473-3099(06)70466-2 PMC7106411 16631551

[4] Chien Y, Klugman K, Morens D (2009). Bacterial pathogens and death during the 1918 influenza pandemic. New Engl J Med.

[5] Colamussi M, White M, Crouch E, Hartshorn K (1999). Influenza A virus accelerates neutrophil apoptosis and markedly potentiates apoptotic effects of bacteria. Blood.

[6] Engelich G, White M, Hartshorn K (2001). Neutrophil survival is markedly reduced by incubation with influenza virus and $${{S}treptococcus pneumoniae}$$: Role of respiratory burst. J Leukoc Biol.

[7] Ghoneim H, McCullers J (2014). Adjunctive corticosteroid therapy improves lung immunopathology and survival during severe secondary pneumococcal pneumonia in mice. J Infect Dis.

[8] Ghoneim H, Thomas P, McCullers J (2013). Depletion of alveolar macrophages during influenza infection facilitates bacterial superinfections. J Immunol.

[9] Gubareva L, Kaiser L, Hayden F (2000). Influenza virus neuraminidase inhibitors. Lancet.

[10] Hamilton J (2008). Colony-stimulating factors in inflammation and autoimmunity. Nat Rev Immunol.

[11] Hayden FG (2013). Newer influenza antivirals, biotherapeutics and combinations. Influenza Other Respir Viruses.

[12] Karlström Å, Boyd K, English B, McCullers J (2009). Treatment with protein synthesis inhibitors improves outcomes of secondary bacterial pneumonia after influenza. J Infect Dis.

[13] Karlström Å, Heston S, Boyd K, Tuomanen E, McCullers J (2011). Toll-like receptor 2 mediates fatal immunopathology in mice during treatment of secondary pneumococcal pneumonia following influenza. J Infect Dis.

[14] Kobayashi S, Braughton K, Whitney A, Voyich J, Schwan T, Musser J, DeLeo F (2003) Bacterial pathogens modulate an apoptosis differentiation program in human neutrophils. P Natl Acad Sci USA 100(19):10948–10953 10.1073/pnas.1833375100 PMC196908 12960399

[15] Li R, Lim A, Phoon M, Narasaraju T, Ng J, Poh W, Sim M, Chow V, Locht C, Alonso S (2010). Attenuated Bordetella pertussis protects against highly pathogenic influenza A viruses by dampening the cytokine storm. J Virol.

[16] Liu X, He Y, Xiao K, White J, Fusco D, Papanicolaou G (2013) Effect of linezolid on clinical severity and pulmonary cytokines in a murine model of influenza A and* Staphylococcus aureus* coinfection. PloS One 8(3):e57483 10.1371/journal.pone.0057483 PMC3589409 23478252

[17] Louria D, Blumenfeld H, Ellis J, Kilbourne E, Rogers D (1959) Studies on influenza in the pandemic of 1957–1958. II. Pulmonary complications of influenza. J Clin Investig 38(1 Pt 1-2):213–265 10.1172/JCI103791 PMC444127 13620784

[18] McCullers J (2004). Effect of antiviral treatment on the outcome of secondary bacterial pneumonia after influenza. J Infect Dis.

[19] McCullers J (2011). Preventing and treating secondary bacterial infections with antiviral agents. Antivir Ther.

[20] McCullers J (2014). The co-pathogenesis of influenza viruses with bacteria in the lung. Nat Rev Microbiol.

[21] McCullers J, Bartmess K (2003). Role of neuraminidase in lethal synergism between influenza virus and $${{S}treptococcus pneumoniae}$$. J Infect Dis.

[22] McCullers J, Rehg J (2002). Lethal synergism between influenza virus and $${{S}treptococcus pneumoniae}$$: characterization of a mouse model and the role of platelet-activating factor receptor. J Infect Dis.

[23] McNamee L, Harmsen A (2006). Both influenza-induced neutrophil dysfunction and neutrophil-independent mechanisms contribute to increased susceptibility to a secondary $${{S}treptococcus pneumoniae}$$ infection. Infect Immun.

[24] Metzger D, Sun K (2013). Immune dysfunction and bacterial coinfections following influenza. J Immunol.

[25] Metzger D, Furuya Y, Salmon S, Roberts S, Sun K (2015). Limited efficacy of antibacterial vaccination against secondary serotype 3 pneumococcal pneumonia following influenza infection. J Infect Dis.

[26] Mifsud E, Tan A, Short K, Brown L, Chua B, Jackson D (2016). Reducing the impact of influenza-associated secondary pneumococcal infections. Immunol Cell Biol.

[27] Mina M, Klugman K, McCullers J (2013) LAIV, but not PCV, protects against increased density and duration of pneumococcal carriage following influenza infection in pneumococcal colonized mice. J Infect Dis 208(8): 1281–1285 (p jit317) 10.1093/infdis/jit317 PMC6281400 23852122

[28] Mina M, Brown L, Klugman K (2015). Dynamics of increasing IFN-$$\gamma $$ exposure on murine MH-S cell-line alveolar macrophage phagocytosis of $${Streptococcus pneumoniae}$$. J Interf Cytok Res.

[29] Morens D, Taubenberger J, Fauci A (2008). Predominant role of bacterial pneumonia as a cause of death in pandemic influenza: implications for pandemic influenza preparedness. J Infect Dis.

[30] Peltola V, McCullers J (2004) Respiratory viruses predisposing to bacterial infections: role of neuraminidase. Pediatr Infect Dis 23(1, Supplement): S87–S97 10.1097/01.inf.0000108197.81270.35 14730275

[31] Plot digitizer. URL http://plotdigitizer.sourceforge.net/

[32] Reed C, Chaves S, Kirley P, Emerson R, Aragon D, Hancock E, Butler L, Baumbach J, Hollick G, Bennett N, et al (2015) Estimating influenza disease burden from population-based surveillance data in the United States. PLoS One 10(3):e0118,369 10.1371/journal.pone.0118369 PMC4349859 25738736

[33] Robinson K, Kolls J, Alcorn J (2015). The immunology of influenza virus-associated bacterial pneumonia. Curr Opin Immunol.

[34] Rynda-Apple A, Robinson K, Alcorn J (2015). Influenza and bacterial superinfection: illuminating the immunologic mechanisms of disease. Infect Immun.

[35] Short K, Habets M, Hermans P, Diavatopoulos D (2012). Interactions between $${{S}treptococcus pneumoniae}$$ and influenza virus: a mutually beneficial relationship?. Future Microbiol.

[36] Small C, Shaler C, McCormick S, Jeyanathan M, Damjanovic D, Brown E, Arck P, Jordana M, Kaushic C, Ashkar A (2010). Influenza infection leads to increased susceptibility to subsequent bacterial superinfection by impairing NK cell responses in the lung. J Immunol.

[37] Smith A, McCullers J (2014) Secondary bacterial infections in influenza virus infection pathogenesis. In: Influenza pathogenesis and control-volume I, Springer, New York, pp 327–356 10.1007/82_2014_394 PMC7122299 25027822

[38] Smith A, Smith A (2016) A critical, nonlinear threshold dictates bacterial invasion and initial kinetics during influenza. bioRxiv p 052175 10.1038/srep38703 PMC5156930 27974820

[39] Smith M, Schmidt J, Rehg J, Orihuela C, McCullers J (2007). Induction of pro-and anti-inflammatory molecules in a mouse model of pneumococcal pneumonia after influenza. Comp Med.

[40] Smith A, Adler F, Perelson A (2010). An accurate two-phase approximate solution to an acute viral infection model. J Math Biol.

[41] Smith A, Adler F, McAuley J, Gutenkunst R, Ribeiro R, McCullers J, Perelson A (2011a) Effect of 1918 PB1-F2 expression on influenza A virus infection kinetics. PLoS Comput Biol 7(2):e1001081 10.1371/journal.pcbi.1001081 PMC3040654 21379324

[42] Smith A, McCullers J, Adler F (2011b) Mathematical model of a three-stage innate immune response to a pneumococcal lung infection. J Theor Biol 276(1):106–116. doi:10.1016/j.jtbi.2011.01.052 10.1016/j.jtbi.2011.01.052 PMC3066295 21300073

[43] Smith A, Adler F, Ribeiro R, Gutenkunst R, McAuley J, McCullers J, Perelson A (2013) Kinetics of coinfection with influenza A virus and *Streptococcus pneumoniae*. PLoS Pathog 9(3):e1003238–e1003238 10.1371/journal.ppat.1003238 PMC3605146 23555251

[44] Sun K, Metzger D (2008). Inhibition of pulmonary antibacterial defense by interferon-$$\gamma $$ during recovery from influenza infection. Nat Med.

[45] Thompson W, Shay D, Weintraub E, Brammer L, Cox Bridges N CB, Fukuda K (2004). Influenza-associated hospitalizations in the United States. J Am Med Assoc.

[46] Warnking K, Klemm C, Löffler B, Niemann S, Krüchten A, Peters G, Ludwig S, Ehrhardt C (2015). Super-infection with $${{S}taphylococcus aureus}$$ inhibits influenza virus-induced type I IFN signalling through impaired STAT1-STAT2 dimerization. Cell Microbiol.

[47] Weeks-Gorospe J, Hurtig H, Iverson A, Schuneman M, Webby R, McCullers J, Huber V (2012). Naturally occurring swine influenza A virus PB1-F2 phenotypes that contribute to superinfection with gram-positive respiratory pathogens. J Virol.

[48] Weinberger D, Simonsen L, Jordan R, Steiner C, Miller M, Viboud C (2012). Impact of the 2009 influenza pandemic on pneumococcal pneumonia hospitalizations in the United States. J Infect Dis.

