# Supplementary material for: Quantifying the therapeutic requirements and potential for combination therapy to prevent bacterial coinfection during influenza
Source: J Pharmacokinet Pharmacodyn. 2016 Sep 27;44(2):81–93. doi: 10.1007/s10928-016-9494-9 (PMC5376398; doi:10.1007/s10928-016-9494-9)
Supplement: Supplementary file 1 — Supplementary material 1 (pdf 8392 KB) [file 10928_2016_9494_MOESM1_ESM.pdf]

Supplementary Information

Quantifying the Therapeutic Requirements and Potential for Combination Therapy to Prevent Bacterial Coinfection During Influenza

Amber M. Smith*

[1]Department of Infectious Diseases, St. Jude Children's Research Hospital, Memphis, TN 38105, USA

*Email: amber.smith@stjude.org

Schematic and Fits of the Influenza Infection Model

The dynamics of the influenza virus infection model (Equations (1)–(4) in the main text) are illustrated in Figure S1A. The model fits to lung viral titers of groups of mice infected with influenza A/Puerto Rico/8/34 (H1N1) (PR8) are shown in Figure S1B for the model parameters in Table 1 (main text). See the main text and Smith et al (2011) for further description of the model and data.

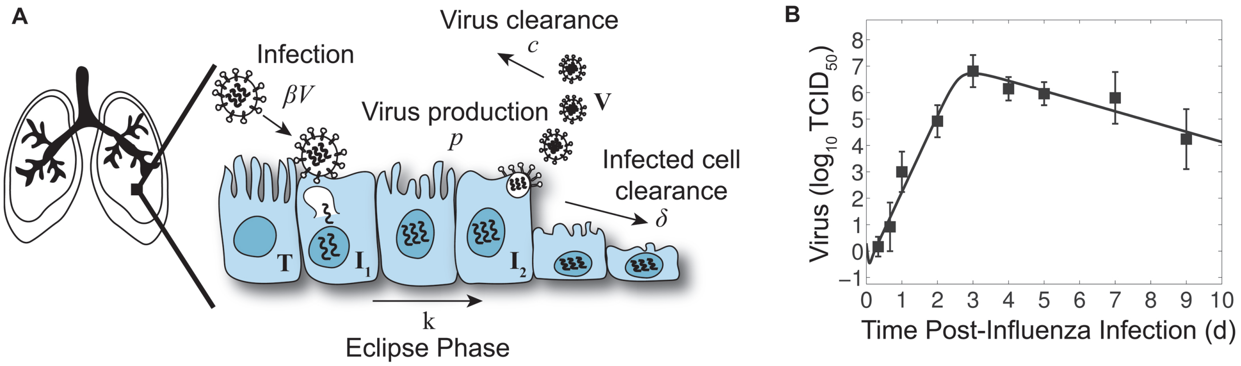

Figure S1: Schematic and Fits of the Virus Infection Model (Smith et al, 2011). (A) Schematic of the virus infection model in Equations (1)–(4). Target cells (T) are infected with virus (V) at rate βV. Infected cells enter an eclipse phase (I_1) and transition to producing virus (I_2) at rate k. Productive infected cells (I_2) produce virus at rate p. Infected cells are removed at rate δ. (B) Fit of the virus infection model (Equations (1)–(4) (main text)) to viral lung titers of mice infected with 100 $TCID_{50}$ PR8 (black squares). The solid black line is the model solution for the parameters in Table 1 (main text).

Schematic and Fits of the Influenza–Pneumococcal Coinfection Model

The dynamics of the coinfection infection model (Equations (5)–(9) in the main text) are illustrated in Figure S2A. The model fits to lung viral and bacterial titers of groups of mice infected with PR8 followed by pneumococcal strain D39 at 7d post-influenza infection (pii) are shown in Figure S2B–C for the model parameters in Table 1 (main text). See the main text and Smith et al (2013) for further description of the model and data.

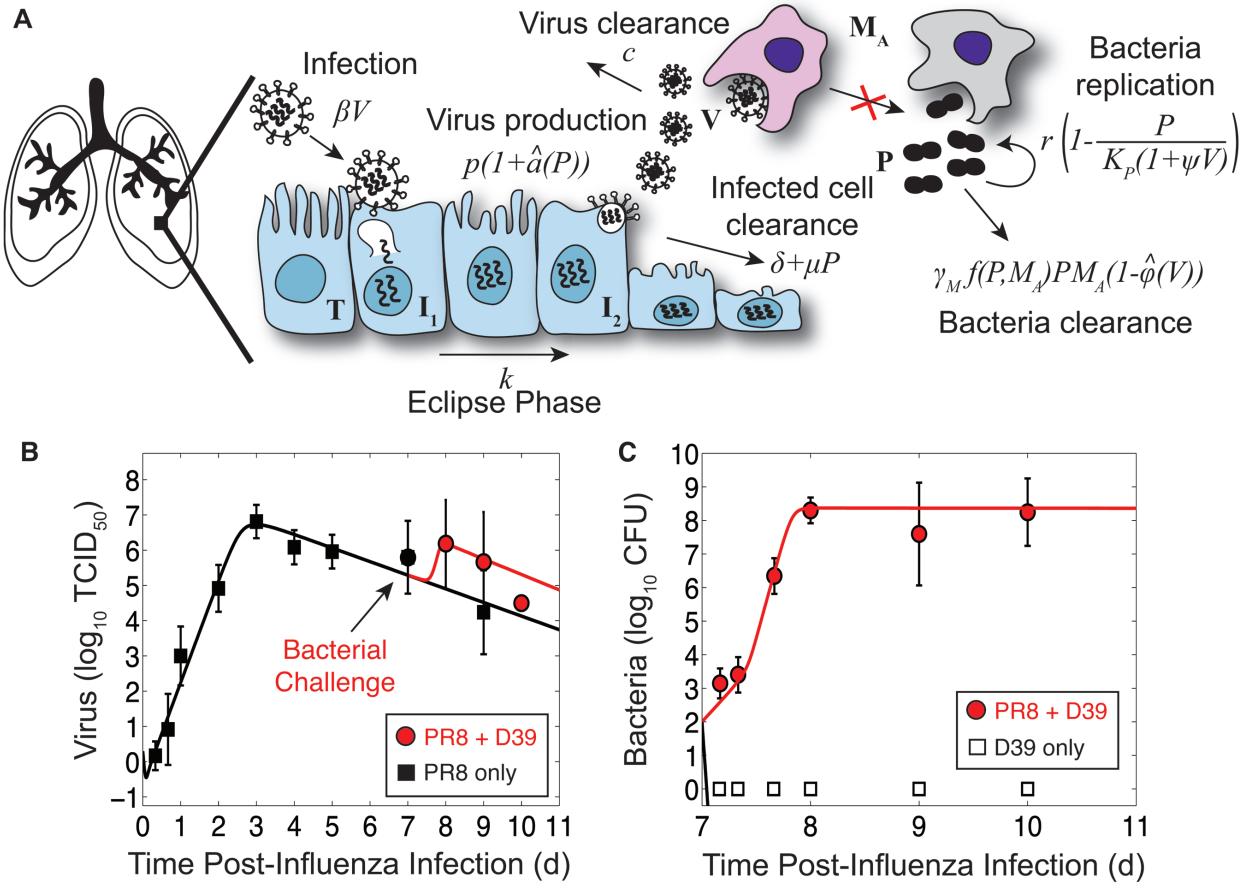

Figure S2: Schematic and Fits of the Coinfection Model (Smith et al, 2013). (A) Schematic of the coinfection model in Equations (5)–(9). Target cells (T) are infected with virus (V) at rate βV. Infected cells enter an eclipse phase (I_1) and transition to producing virus (I_2) at rate k. Productive infected cells (I_2) produce virus at rate p, which is increased by $\hat{a}(P) = aP^z$ when bacteria are present. Infected cells are removed at rate δ and are killed by bacteria at rate μP. Bacteria (P) replicate logistically with maximal rate r and carrying capacity K_P, which is increased by ψV when virus is present. Bacteria are phagocytosed by alveolar macrophages (M_A) at rate $\gamma_M f(P, M_A)$, which is decreased by $\hat{\phi}(V) = \phi V/(K_{PV} + V)$ when virus is present. (B–C) Fit of the coinfection model (red lines, Equations (5)–(9) (main text)) to viral and bacterial lung titers of mice infected with 100 $TCID_{50}$ PR8 followed by 1000 CFU D39 at 7d pii (red circles). The solid black and white squares are data in the absence of bacteria (Panel B) or virus (Panel C), respectively. The solid black lines are the model solution for the parameters in Table 1 (main text) with $P = 0$ (Panel B) or $V = 0$ (Panel C).

Correlation Between RLU and CFU

To establish a correlation between colony forming units (CFU) and relative light units (RLU) over a large range, lung bacterial titers at 4, 8, 24, 48, or 72h post-bacterial infection (pbi) were obtained from mice infected with 1000 CFU D39 7d after infection with 100 $TCID_{50}$ PR8 (see Methods below). The data and the linear fit to the $\log_{10}$–$\log_{10}$ data are shown in Figure S3. The linear fit describing the correlation is given by Equation (13) (main text).

Methods

Use of Experimental Animals

All experimental procedures were approved by the Animal Care and Use Committee at SJCRH under relevant institutional and American Veterinary Medical Association guidelines and were performed in a Biosafety level 2 facility that is accredited by AALAAS.

Mice

Adult (6 week old) female BALB/cJ mice were obtained from Jackson Laboratories (Bar Harbor, ME). Mice were housed in groups of five mice in high-temperature 31.2cm × 23.5cm × 15.2cm polycarbonate cages with isolator lids. Rooms used for housing mice were maintained on a 12:12-hour light:dark cycle at 22 ± 2°C with 50% humidity in the biosafety level 2 facility at St. Jude Children's Research Hospital (Memphis, TN). Prior to inclusion in the experiments, mice were allowed at least 7 days to acclimate to the animal facility such that they were 7 weeks old at the time of infection. Laboratory Autoclavable Rodent Diet (PMI Nutrition International, St. Louis, MO) and autoclaved water were available ad libitum. All experiments were performed under an approved protocol and in accordance with the guidelines set forth by the Animal Care and Use Committee at St. Jude Children's Research Hospital.

Infection Experiments

Experiments were conducted using the mouse adapted influenza PR8 and type 2 pneumococcal strain D39 that was transformed with the lux operon (Xenogen) to make it bioluminescent (McCullers and Bartmess (2003)). The viral infectious dose ($TCID_{50}$) was determined by interpolation using the method of Reed and Muench (1938) using serial dilutions of virus on Madin-Darby canine kidney (MDCK) cells. The bacterial infectious dose (CFU) was counted for serial dilutions of bacteria on tryptic soy-agar plates supplemented with 3% (vol/vol) sheep erythrocytes. Inocula were diluted in sterile PBS and administered intranasally to groups of five mice lightly anesthetized with 2.5% inhaled isoflurane (Baxter, Deerfield, IL) in a total volume of 100ul (50ul per nostril). Mice were inoculated with 100 $TCID_{50}$ PR8 at day 0 followed by 1000 CFU D39 at day 7. Mice were weighed at the onset of infection and each subsequent day for illness and mortality. Mice were euthanized if they became moribund or lost 30% of their starting body weight.

Imaging of Live Mice

To measure the bacterial loads (RLU) in the lungs of live mice, mice were imaged for 60s using an IVIS CCD camera (Caliper Life Sciences, Alameda, CA) after pneumococcal challenge and prior to euthanasia. Total photon emission from a 10.8 cm^2 area of the thorax of each mouse was quantified using LivingImage software (Caliper Life Sciences) as described previously (Francis et al, 2001). Data are expressed as the flux of light per min.

Lung Titers

After imaging, mice were euthanized by CO_2 asphyxiation. Lungs were aseptically harvested, washed three times in PBS, and placed in 500ul PBS. Lungs were mechanically homogenized using an Ultra-Turrax T8 homogenizer (IKA-werke, Staufen, Germany). Lung homogenates were pelleted at 10,000 rpm for 5 min and the supernatants were used to determine the bacterial titer (CFU) for each set of lungs using serial dilutions on tryptic soy-agar plates supplemented with 3% (vol/vol) sheep erythrocytes.

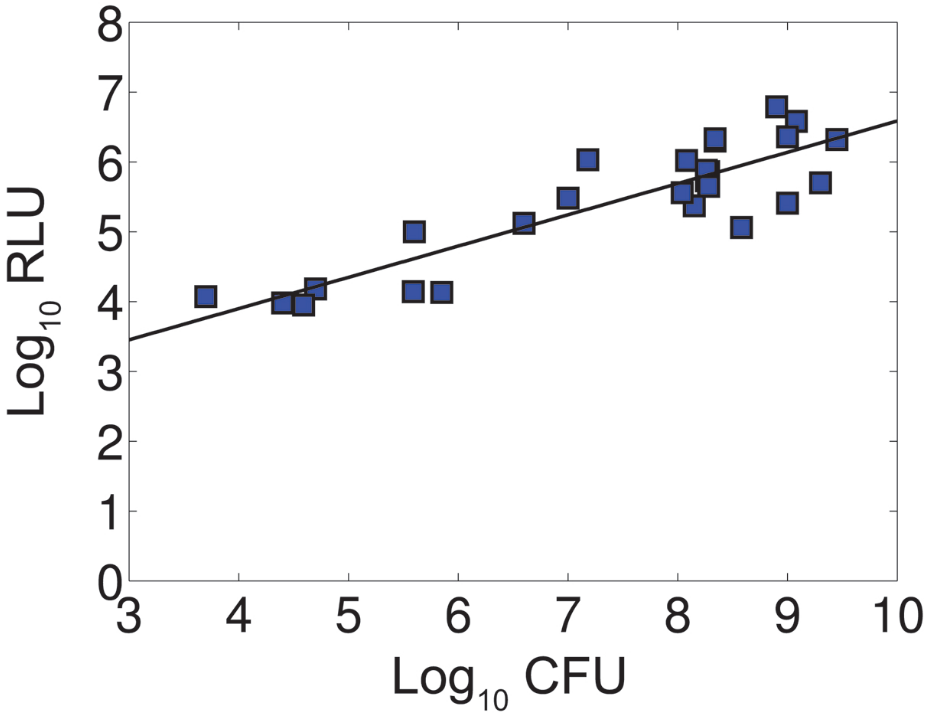

Figure S3: Correlation Between RLU and CFU. Lung bacterial titers ($\log_{10}$ RLU versus $\log_{10}$ CFU, blue squares) from mice infected with 1000 CFU D39 7d after infection with 100 $TCID_{50}$ PR8. The black line is the linear fit to these data and is defined by Equation (13) (main text).

References

Francis KP, Yu J, Bellinger-Kawahara C, Joh D, Hawkinson MJ, Xiao G, Purchio TF, Caparon MG, Lipsitch M, Contag PR (2001) Visualizing Pneumococcal Infections in the Lungs of Live Mice Using Bioluminescent *Streptococcus pneumoniae* Transformed with a Novel Gram-Positive lux Transposon. Infect Immun 69(5):3350–3358

McCullers JA, Bartmess KC (2003) Role of neuraminidase in lethal synergism between influenza virus and *Streptococcus pneumoniae*. J Infect Dis 187(6):1000–1009

Reed LJ, Muench H (1938) A simple method of estimating fifty percent endpoints. Am J Epidemiol 27(3):493–497

Smith AM, Adler FR, McAuley JL, Gutenkunst RN, Ribeiro RM, McCullers JA, Perelson AS (2011) Effect of 1918 PB1-F2 expression on influenza A virus infection kinetics. PLoS Comput Biol 7(2):e1001,081

Smith AM, Adler FR, Ribeiro RM, Gutenkunst RN, McAuley JL, McCullers JA, Perelson AS (2013) Kinetics of coinfection with influenza A virus and *Streptococcus pneumoniae*. PLoS Pathog 9(3):e1003,238–e1003,238
